# Pelvic Malignant Tumor with Liver Metastasis is Actually Conventional Leiomyoma with Abdominal Metastasis

**DOI:** 10.70352/scrj.cr.25-0157

**Published:** 2025-08-14

**Authors:** Jingjun Yang, Jianming Lu

**Affiliations:** Department of General Surgery, Haining People’s Hospital, Haining, Jiaxing, Zhejiang, China

**Keywords:** uterine fibroids, frozen section, overtreatment

## Abstract

**INTRODUCTION:**

For this case, when the preoperative diagnosis indicated malignant tumor with metastasis, intraoperative frozen section examination was performed to adjust the surgical plan accordingly. This approach helped avoid overtreatment, minimizing the patient’s pain and surgical trauma. This case holds educational significance.

**CASE PRESENTATION:**

A 45-year-old female patient underwent open myomectomy 12 years ago for uterine fibroids and laparoscopic subtotal hysterectomy 10 years ago for the same condition. During this check-up, her CA125 was found to be elevated. Further examinations, including ultrasound, enhanced CT, and enhanced MRI, all suggested a pelvic malignant tumor with liver metastasis. The patient underwent tumor resection, and both intraoperative frozen section and routine histopathologic examination confirmed that the pelvic and subphrenic tumors (which had been considered as liver metastases preoperatively) were both conventional leiomyomas.

**CONCLUSIONS:**

This case highlights that leiomyomas, when metastatic, are easily misdiagnosed as malignant tumors with metastasis, presenting a significant challenge for preoperative diagnosis. Clinicians should maintain a high level of suspicion in such cases to avoid overtreatment. In this case, the intraoperative frozen section played a crucial role in preventing unnecessary pelvic lymph node dissection.

## Abbreviations

CA125
: carbohydrate antigen 125
LPD
: leiomyomatosis peritonealis disseminata
MMT
: mesothelial-to-mesenchymal transition
TCT
: ThinPrep cytologic test

## INTRODUCTION

Uterine leiomyoma is the most common benign tumor of the female reproductive tract, with a prevalence of approximately 20%–30% in women of reproductive age.^1)^ Typical symptoms include menorrhagia, pelvic pressure, and chronic pain. Although usually confined to the uterus, some leiomyomas may undergo ectopic implantation, posing diagnostic challenges. Ectopic leiomyomas include parasitic leiomyomas and LPD.^2)^ The proposed mechanisms involve iatrogenic dissemination, intravascular spread, and MMT.^3)^ Patients with a history of myomectomy or laparoscopic surgery are at higher risk.^4)^ Imaging often reveals solitary or multiple solid masses, which can be misdiagnosed as ovarian cancer or peritoneal carcinomatosis.^5)^ A comprehensive preoperative assessment of history, imaging, and biomarkers is critical for accurate diagnosis. CA125 is widely used in ovarian cancer screening but lacks specificity. CA125 testing is not recommended as the sole factor for differentiating between a benign and a malignant adnexal mass.^6)^ This case highlights the rare coexistence of pelvic leiomyoma with subdiaphragmatic implantation, underscoring the pivotal role of intraoperative frozen section analysis in achieving an accurate diagnosis.

## CASE PRESENTATION

The patient is a 45-year-old female; 12 years ago, she underwent an open myomectomy, and 10 years ago, she had a laparoscopic subtotal hysterectomy for uterine fibroids. She was asymptomatic at the time of this examination, but her CA125 level was elevated at 49.9 U/mL.

**Fig. 1:**
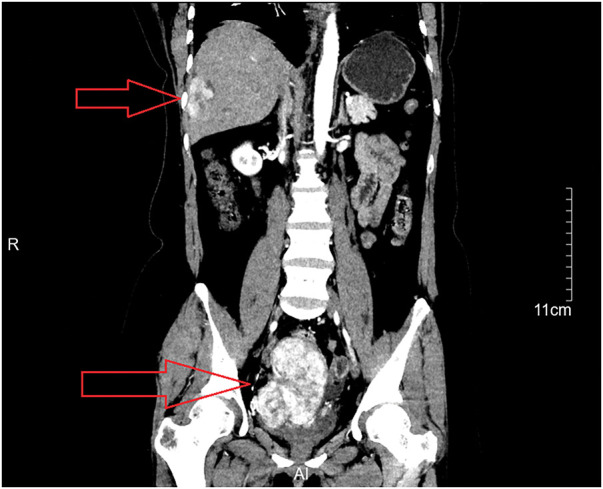
CT coronal view shows a pelvic tumor and subphrenic metastases.

Further examination results are as follows:

- Urinalysis and stool routine tests: No abnormalities were detected.
- Cervical TCT: Negative for intraepithelial lesion or malignancy.
- Bilateral breast and axillary ultrasound: Multiple nodules were found in both breasts; the largest on the left measured 5.6 × 3.5 mm (BI-RADS category 3), and the largest on the right measured 9.1 × 3.0 mm (BI-RADS category 3).
- Bilateral mammography: Glandular hyperplasia was noted in both breasts. Calcification was observed in the right breast (BI-RADS category 2), and a nodule was identified in the left breast (BI-RADS category 3).
- Ultrasound of the liver, gallbladder, pancreas, spleen, ureters, and bladder: A space-occupying lesion measuring 53 × 30 mm was found in the liver. No abnormalities were detected in the gallbladder, pancreas, spleen, kidneys, ureters, or bladder.
- Transvaginal ultrasound: A heterogeneous hypoechoic solid mass in the pelvis.
- Contrast-enhanced MRI of the uterus and adnexa: Post-subtotal hysterectomy status, with a right-sided pelvic mass suspected to be a malignant tumor; multiple nabothian cysts were observed in the residual cervix.
- Contrast-enhanced CT of the whole abdomen: Post-subtotal hysterectomy status, with a mass in the right adnexal region and a subcapsular hepatic lesion in the right liver lobe, suggesting a malignant tumor in the right adnexa with liver metastasis. Multiple cystic lesions were also observed in both adnexal regions (**Fig. 1**).

The patient was preoperatively diagnosed with a pelvic malignant tumor with liver metastasis. Differential diagnoses included ovarian cancer, primary peritoneal carcinoma, cervical cancer, colorectal cancer, gastric cancer, bladder cancer, and breast cancer.

The cervical TCT was negative, making cervical cancer highly unlikely. No red blood cells were detected in the urine, and bladder ultrasound revealed no abnormalities, thus bladder cancer was not supported. Breast ultrasound and mammography did not reveal any malignant nodules, making breast cancer an unlikely diagnosis.

The patient reported no upper abdominal bloating or discomfort, no epigastric pain, belching, acid reflux, nausea, vomiting, or melena. Stool tests showed no red blood cells or occult blood. Additionally, contrast-enhanced CT revealed no obvious masses in the gastric wall, suggesting that the likelihood of gastric cancer with metastasis is low. However, it is regrettable that the patient did not undergo gastroscopy prior to surgery to fully exclude gastric cancer.

The patient did not experience symptoms such as increased bowel frequency, tenesmus, anal pressure, anal pain, or blood/mucus in the stool. Routine stool tests showed no red blood cells or occult blood. These findings do not support a diagnosis of colorectal cancer. Nevertheless, the patient did not undergo digital rectal examination or colonoscopy, and the pelvic tumor was located near the colorectum. Therefore, colorectal cancer with liver metastasis could not be entirely ruled out.

Ovarian cancer and primary peritoneal carcinoma were considered the most likely diagnoses. The patient’s clinical presentation was highly consistent with these conditions. Based on the comprehensive analysis, the primary diagnostic considerations were as follows:

- Ovarian cancer with liver metastasis;
- Primary peritoneal carcinoma with liver metastasis;
- Colorectal cancer with liver metastasis.

The patient and family were informed of the condition, and the patient consented to undergo open tumor resection surgery.

**Fig. 2:**
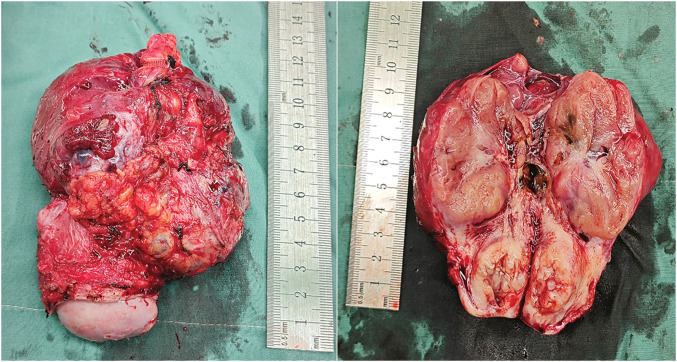
Resected pelvic tumor.

**Fig. 3:**
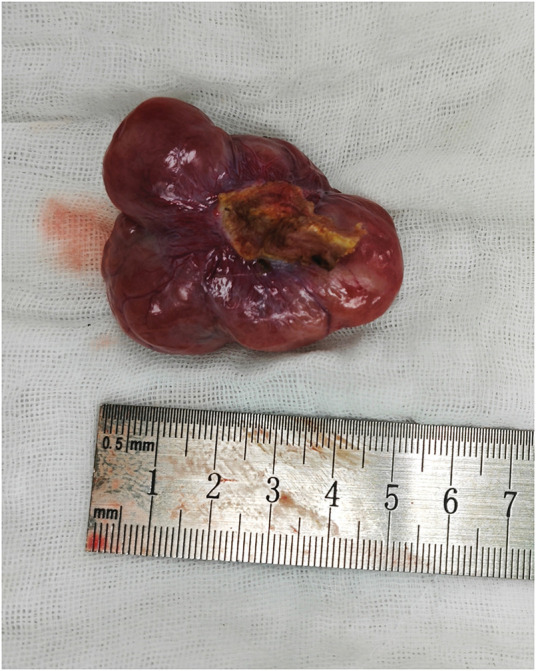
Resected subdiaphragmatic tumor.

Intraoperatively, the pelvic tumor was found to be inseparable from the residual cervix and right adnexa. A gynecologic consultation was sought, and a decision was made to remove the pelvic tumor, right adnexa, and residual cervix together (**Fig. 2**). During intraoperative exploration of the suspected “liver metastasis,” it was found that the lesion was actually a pedunculated subserosal tumor resulting from peritoneal implantation beneath the right diaphragm. The tumor was subsequently resected (**Fig. 3**). Both tumors had intact capsules and smooth surfaces, and based on intraoperative observations, their malignancy potential was considered low. Therefore, a frozen section examination was performed, which revealed the following:[Fig F4][Fig F5]

**Fig. 4 F4:**
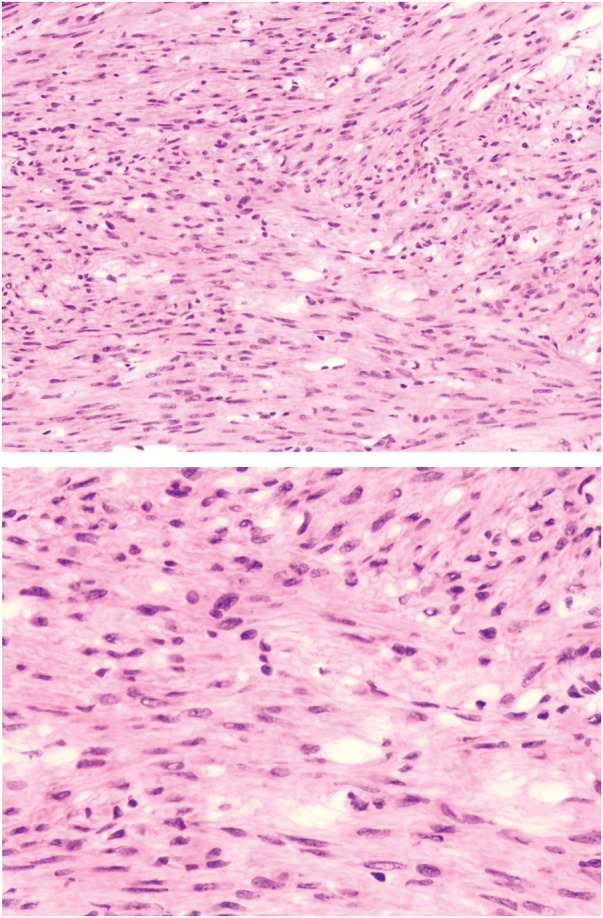
Intraoperative frozen section image of the pelvic tumor.

**Fig. 5 F5:**
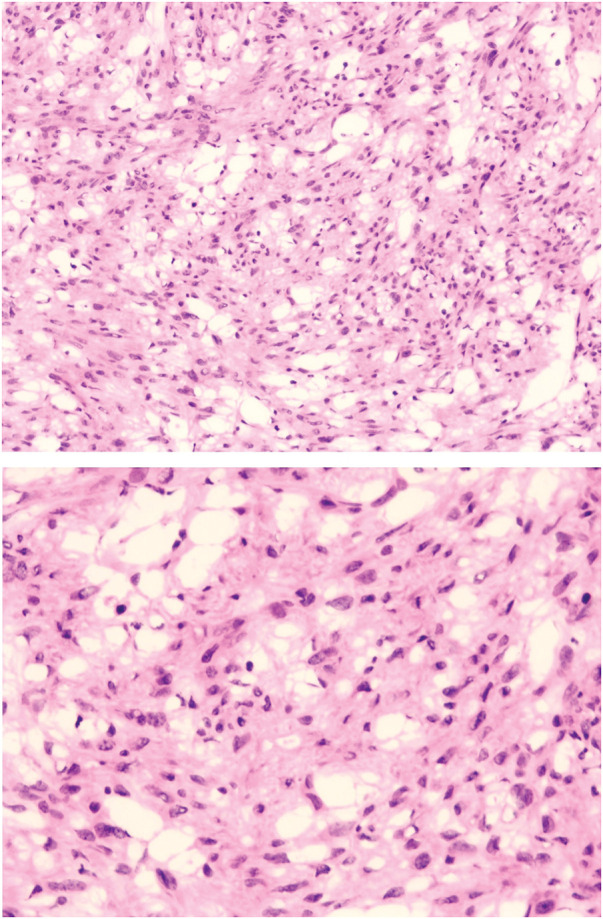
Intraoperative frozen section image of the subdiaphragmatic tumor.

- Pelvic tumor: Suggestive of a leiomyoma (**Fig. 4**).
- Subdiaphragmatic tumor: Spindle cell tumor, favoring leiomyoma (**Fig. 5**).

A definitive intraoperative diagnosis of pelvic leiomyoma with subdiaphragmatic implantation metastasis was established. Consequently, pelvic lymph node dissection was deemed unnecessary. The surgery was successfully completed, and the patient had an uneventful recovery without complications, leading to a smooth discharge.

Postoperative routine histopathologic report:[Fig F6][Fig F7]

**Fig. 6 F6:**
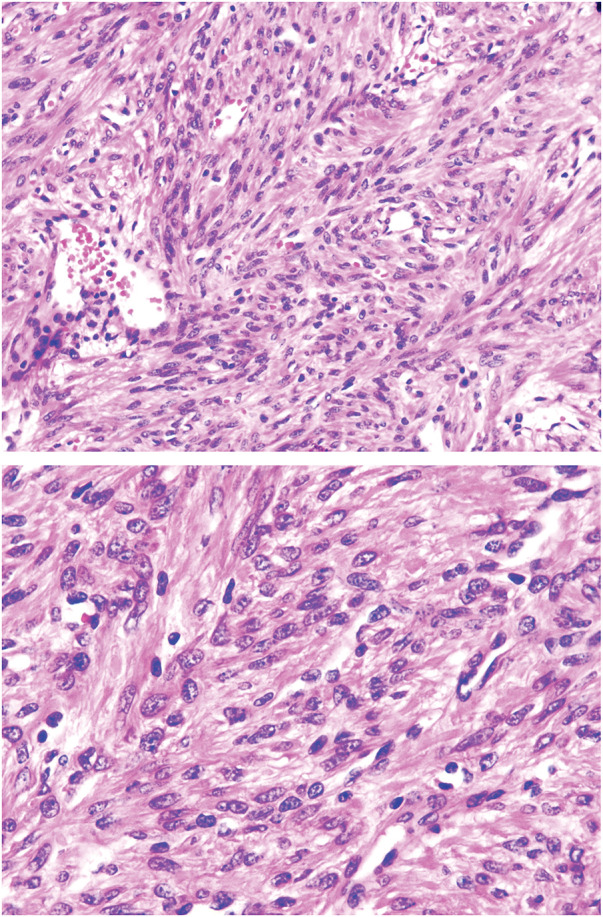
Pathological images of the pelvic tumor.

**Fig. 7 F7:**
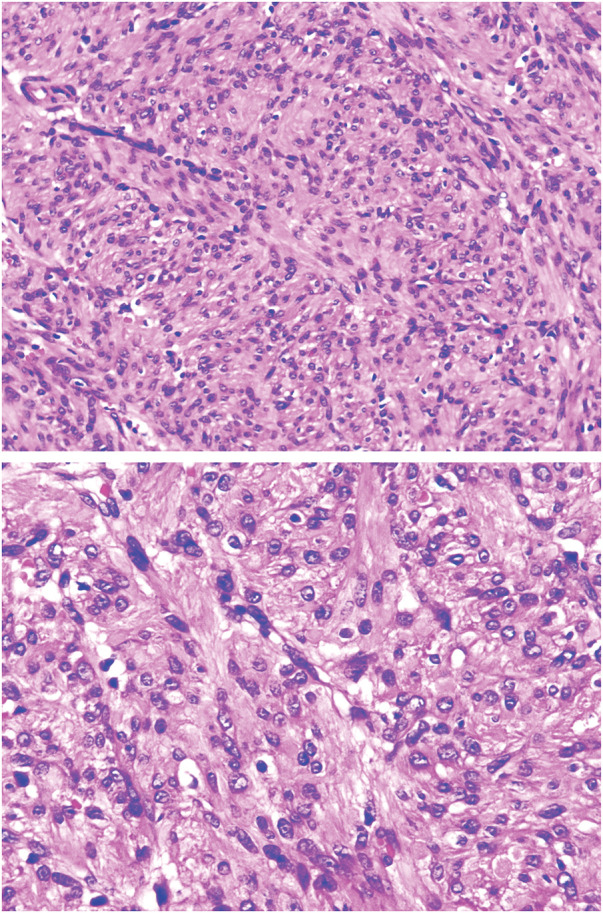
Pathological images of the subdiaphragmatic tumor.

1. Pelvic tumor: Spindle cell tumor. Based on morphology and immunohistochemical findings, the diagnosis is consistent with a conventional leiomyoma (**Fig. 6**).
2. Subdiaphragmatic tumor: Spindle cell tumor. Based on morphology and immunohistochemical findings, the diagnosis is consistent with a conventional leiomyoma (**Fig. 7**).

One month postoperatively, follow-up testing showed a decrease in CA125 levels to 23.4 U/mL.

## DISCUSSION

Pathogenesis of leiomyoma metastasis: While conventional leiomyomas are benign, rare cases of peritoneal implantation have been reported, raising questions about the mechanisms underlying this phenomenon.

- Mechanical seeding hypothesis: Some studies suggest that mechanical seeding during surgical procedures, such as myomectomy or hysterectomy, may lead to the dispersion of leiomyoma cells within the peritoneal cavity, facilitating implantation.^7)^
- Hematogenous and lymphatic spread: Although rare, some researchers hypothesize that leiomyoma cells might enter the bloodstream or lymphatic system, leading to distant implantation.^8)^
- Molecular and genetic alterations: Certain genetic mutations, such as *MED12* mutations, have been implicated in leiomyoma development. It is possible that specific mutations contribute to an increased capacity for implantation and growth outside the uterus.^9)^
- Hormonal influence: Estrogen and progesterone are known to play a crucial role in leiomyoma growth. Altered hormonal environments in post-surgical or peritoneal conditions may facilitate implantation and proliferation of leiomyoma cells.^10)^

The patient had a history of undergoing 2 surgeries for uterine fibroids, and both leiomyomas were located within the abdominal cavity, which is highly consistent with the Mechanical Seeding Hypothesis.

The pathological diagnosis of this patient confirmed a conventional leiomyoma, a benign tumor, but with peritoneal implantation metastasis. The occurrence of metastasis in benign tumors is uncommon and often leads to a preoperative misdiagnosis of malignancy, as metastasis is typically considered a hallmark of malignant tumors. In cases where a benign tumor exhibits metastatic behavior, distinguishing it from a malignant tumor before surgery becomes highly challenging. In this case, preoperative tumor markers and imaging studies all suggested a malignant pelvic tumor with liver metastasis. However, intraoperative frozen-section pathology confirmed that both the pelvic tumor and the subphrenic lesion (initially suspected as a liver metastasis) were conventional leiomyomas. This accurate intraoperative diagnosis prevented unnecessary pelvic lymphadenectomy, thereby reducing surgical risk, shortening operative time, and minimizing postoperative complications.

## CONCLUSIONS

This case was ultimately diagnosed as parasitic leiomyoma, which was initially misinterpreted as a malignant tumor with metastasis. It highlights the importance of considering benign tumor implantation when evaluating pelvic tumors with abdominal metastases. If intraoperative findings are inconsistent with the preoperative diagnosis, immediate frozen-section pathology should be performed to avoid overtreatment and enhance surgical decision-making precision.

## ACKNOWLEDGMENTS

I sincerely thank Dr. Jun Lu and Dr. Hailong Jin for their invaluable support and assistance.

## DECLARATIONS

### Funding

The authors received no specific funding for this work.

### Authors’ contributions

JY performed the surgical procedures and contributed to the discussion and preparation of the manuscript.

JL contributed to the discussion and preparation of the figures.

All authors have read and approved the manuscript.

### Availability of data and materials

Not applicable.

### Ethics approval and consent to participate

This work does not require ethical considerations or approval. Informed consent to participate in this study was obtained from the patient.

### Consent for publication

Informed consent for publication of this case report was obtained from the patient.

### Competing interest

The authors declare that there is no competing interest.

## References

[1] Stewart EA. Uterine fibroids. Lancet 2001; 357: 293–8. 11214143 10.1016/S0140-6736(00)03622-9

[2] Kho KA Nezhat C. Parasitic myomas. Obstet Gynecol 2009; 114: 611–5. 19701042 10.1097/AOG.0b013e3181b2b09a

[3] Li J Dai S. Leiomyomatosis peritonealis disseminata: a clinical analysis of 13 cases and literature review. Int J Surg Pathol 2020; 28: 163–8. 31615319 10.1177/1066896919880962

[4] Mabrouk M Arena A Raimondo D Leyomiomatosis peritonealis disseminata associated with ovarian endometriosis in a patient submitted to hysteroscopic myomectomy. Fertil Steril 2019; 111: 1259. 31030890 10.1016/j.fertnstert.2019.03.023

[5] Fasih N Prasad Shanbhogue AK Macdonald DB Leiomyomas beyond the uterus: unusual locations, rare manifestations. Radiographics 2008; 28: 1931–48. 19001649 10.1148/rg.287085095

[6] Biggs WS Marks ST. Diagnosis and management of adnexal masses. Am Fam Physician 2016; 93: 676–81. 27175840

[7] Whalen C Murphy M Timmins P. Disseminated leiomyomatosis following a robotic-assisted myomectomy with power morcellation: a case report. Cureus 2024; 16**: **e75971. 39835060 10.7759/cureus.75971 PMC11742743

[8] Wojtyś ME Kacalska-Janssen O Ptaszyński K Benign metastasizing leiomyoma of the lung: diagnostic process and treatment based on three case reports and a review of the literature. Biomedicines 2022; 10: 2465. 36289727 10.3390/biomedicines10102465 PMC9599094

[9] Mäkinen N Kämpjärvi K Frizzell N Characterization of *MED12*, *HMGA2*, and *FH* alterations reveals molecular variability in uterine smooth muscle tumors. Mol Cancer 2017; 16: 101. 28592321 10.1186/s12943-017-0672-1 PMC5463371

[10] Piórek A Płużański A Wiśniewski P Pulmonary benign metastasizing leiomyoma in a postmenopausal woman: a case report and review of the literature. Diseases 2024; 12: 181. 39195180 10.3390/diseases12080181 PMC11353495

